# Unveiling the Rarity: An Anomaly of the Left Circumflex Artery Supplying Left Ventricular Apex and Presenting With Myocardial Infarction

**DOI:** 10.7759/cureus.68585

**Published:** 2024-09-03

**Authors:** Satyajit Singh, Amratansh Varshney, Abhishek Kumar, Surendra K Naik

**Affiliations:** 1 Cardiology, All India Institute of Medical Sciences, Raipur, Raipur, IND

**Keywords:** interventional management, myocardial infarction, left ventricular apex, left circumflex artery (lcx), anomalous coronary arteries

## Abstract

Coronary arteries are typically identified based on the myocardial territory they supply. In rare cases of coronary artery anomalies, the apex of the heart may be supplied by arteries other than the left anterior descending artery. While it is more common for the posterior descending artery from the right coronary artery to supply the apex, there are rare instances where the left circumflex (LCX) artery performs this function. This case report describes an unusual occurrence where the left ventricular apex is supplied by an obtuse marginal branch of the LCX artery. We present this case due to its rarity, unique presentation, and the challenges it poses for both medical and surgical management.

## Introduction

Coronary artery anomalies (CAAs) involve irregularities in the origin, course, and distribution of coronary vessels. Despite their relatively low prevalence, estimated at around 1% in the general population and 3-5% in patients undergoing coronary angiography, CAAs have significant clinical implications [1]. Often discovered incidentally during angiographic assessments, these anomalies are typically asymptomatic but can lead to life-threatening events, particularly when the anomalous vessels follow a precarious course [2]. Among the notable variations, the left circumflex (LCX) coronary artery originating from the right sinus of Valsalva is a common anatomical anomaly. Although traditionally considered benign, such anomalies can cause myocardial ischemia due to factors such as accelerated atherosclerosis, spasm, ostial stenosis, extrinsic compression, or mechanical distortion from adjacent structures [3]. Accurate delineation of coronary anatomy and identification of high-risk features are crucial for managing patients with CAAs. CT coronary angiography (CCTA) is now the gold standard for noninvasive imaging, providing comprehensive visualization of coronary vasculature [4]. Provocative testing and continuous electrocardiographic monitoring are essential for assessing inducible myocardial ischemia and detecting arrhythmic events, especially when clinical suspicion is high. Cardiac MRI is also gaining importance as an adjunctive tool, particularly when CCTA findings are inconclusive [5].

This paper presents a rare case of an obtuse marginal (OM) branch arising from the LCX with an unusually elongated course supplying the entire left ventricular apex, leading to acute myocardial infarction (MI). This case highlights the need to recognize and thoroughly evaluate coronary anomalies, even in seemingly benign anatomical variations. There are no definitive recommendations for managing CAAs, as treatment must be individualized. Based on clinical case reports, management options may include conservative approaches, percutaneous interventions, or surgical procedures [6]. This report enhances our understanding of how unusual coronary artery structures impact clinical outcomes by examining detailed clinical and angiographic data. Conducting large prospective studies on rare and phenotypically variable conditions presents challenges.

A preprint version of this article was previously posted to the Research Square server on February 29, 2024.

## Case presentation

A 64-year-old woman arrived at the ED with chest pain at rest, radiating to her back, and accompanied by sweating and shortness of breath. She had no known medical conditions or prior history of cardiac issues. On examination, her vital signs were stable, with a heart rate of 90 beats per minute and a right arm blood pressure of 90/62 mmHg. Her oxygen saturation was 99% on room air, and there were no signs of pallor, icterus, clubbing, cyanosis, or pedal edema. A cardiac examination revealed a grade three pansystolic, high-pitched murmur in the mitral area, radiating to the back and increasing in intensity upon standing from a sitting position. A non-ejection click was also heard in the mitral area. Chest auscultation showed vesicular breath sounds with bilateral infra-scapular fine crepitations in the back.

**Figure 1:**
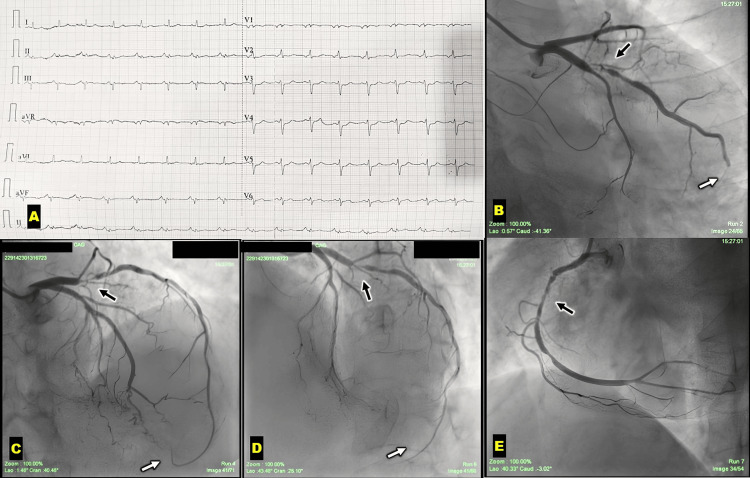
(A) ECG showing sinus rhythm, left axis deviation, Q waves in leads III and aVF, and subtle ST segment depression in leads V3 to V5. (B) CAG in AP caudal projection showing the LCX artery giving rise to a large OM branch. The OM branch exhibits ostio-proximal critical stenosis (black arrow) and extends toward the apex of the LV (white arrow). (C) CAG in AP cranial projection illustrating a type I LAD artery. A large OM branch with ostio-proximal critical stenosis (black arrow) descends to supply the apex of the LV. (D) CAG in LAO cranial projection displaying the LAD, LCX, and OM arteries with similar findings. (E) CAG in LAO projection revealing the RCA with significant stenosis in the mid-RCA (black arrow). CAG, coronary angiogram; LAD, left anterior descending; LAO, left anterior oblique; LCX, left circumflex; LV, left ventricle; OM, obtuse marginal; RCA, right coronary artery

ECG showed regular sinus rhythm, left axis deviation, Q waves in leads III and aVF, and subtle ST segment depression in leads V3 to V5 (Figure 1A). Echocardiography revealed a reduced left ventricular ejection fraction of 45%, with mild hypokinesia of the osterior wall. Mid and apical inferior and inferolateral wall segments were severely hypokinetic. The anterior mitral leaflet was myxomatous, prolapsing into the left atrium and causing severe mitral regurgitation (MR). The right ventricle and right atrium were normal in size, with the right ventricle having normal systolic function and a tricuspid annular plane systolic excursion of 20 mm. Cardiac troponin levels were elevated. Based on these findings, she was diagnosed with acute coronary syndrome and severe MR and was taken to the cath lab for a coronary angiogram.

Coronary angiography was performed via the right radial approach, revealing double vessel disease. The left main vessel was normal. The major OM branch of the LCX artery was a large-sized vessel supplying the left ventricular apex and the inferior surface of the heart. It exhibited ostial-proximal critical stenosis (Figure 1B, 1C, 1D, Video 1). The left anterior descending (LAD) artery followed the anterior interventricular groove and terminated before reaching the apex (Type I LAD) (Figure 1C, 1D, Video 2). The right coronary artery (RCA) was a dominant vessel of average size with diffuse plaques in the proximal and mid segments, showing approximately 70-80% stenosis (Figure 1E, Video 3). The posterior descending artery (PDA) was small in size. She was advised to undergo mitral valve replacement along with coronary artery bypass grafting to the OM and RCA vessels. The procedure was performed successfully without complications, and she is doing well in follow-up.

**Video 1:** CAG in AP caudal projection showing the LCX artery giving rise to a large OM branch. The OM branch exhibits proximal critical stenosis (black arrow) and extends toward the apex of the LV. CAG, coronary angiogram; LCX, left circumflex; LV, left ventricle; OM, obtuse marginal

**Video 2:** CAG in AP cranial projection showing a type I LAD artery. A large OM branch with proximal critical stenosis is observed extending downward to supply the apex of the LV. CAG, coronary angiogram; LAD, left anterior descending; LV, left ventricle; OM, obtuse marginal

**Video 3:** CAG in LAO cranial projection showing the RCA with significant stenosis in the mid-RCA segment. CAG, coronary angiogram; LAO, left anterior oblique; RCA, right coronary artery

## Discussion

CAAs have been documented since the 18th century, with the first comprehensive classification and scientific statement on their significance published in 1969 [7]. CAAs can be categorized into three main types: anomalies of origin, course, and termination. Anomalies of origin include anomalous pulmonary or aortic origins and congenital atresia of the left main artery. Anomalies of course encompass myocardial bridging and coronary aneurysms, while anomalies of termination include coronary arteriovenous fistulas and stenosis [8].

Coronary arteries are classified based on the myocardial regions they supply. The LAD artery supplies the anterior interventricular septum and anterior left ventricular free wall, while the LCX artery nourishes the posterolateral left ventricular free wall. The RCA provides blood to the right ventricular free wall. Typically, the left ventricle apex is supplied by the LAD in 77.7% of cases, with some instances receiving blood from both the LAD and the PDA branch of the RCA in 12.1% of cases.

In rare cases, the apex may be supplied solely by the PDA branch of the RCA, or even more exceptionally, by the LCX or OM artery [9]. In these cases, the LAD is often shorter and does not reach the apex. The patient described here had a significant OM artery from the LCX supplying both the apex and inferior surface of the heart, which is an uncommon finding. This case also involved MI and a myxomatous mitral valve with prolapse and severe MR. The relationship between inflammatory or degenerative changes, atherosclerosis in the anomalous coronary artery, and MI is still uncertain. Conversely, chronic ischemia may contribute to mitral valve prolapse and subsequent severe MR.

Diagnostic and treatment approaches based solely on anatomical findings can be insufficient. Evaluating the ischemic burden is crucial for personalized intervention strategies. Traditionally, invasive coronary angiography was the gold standard for identifying and classifying CAAs, but it has been increasingly supplanted by CCTA due to its limited spatial resolution and lack of three-dimensional (3D) imaging. Recent advancements, including intravascular imaging and fractional flow reserve techniques, offer more precise assessments of intraluminal geometry and physiology [10]. Multidetector CCTA is now considered the gold standard for studying CAAs due to its detailed characterization, 3D reconstruction capabilities, and widespread clinical acceptance. Cardiac MRI with late gadolinium enhancement can detect fibrosis, indicating myocardial ischemia. Stress imaging with dobutamine, vasodilators, or exercise positron emission tomography are additional options requiring further investigation [10].

Coronary aneurysms are rare, and most data on their management come from case series with varying pathogenesis [11]. Surgical intervention is typically preferred for symptomatic cases, while asymptomatic cases present a management dilemma. The debate continues regarding the use of prophylactic antiplatelet or anticoagulant therapy and whether to intervene [12]. Recognizing this anomaly is crucial for accurate graft placement and improved outcomes in surgical interventions.

## Conclusions

Among LCX CAAs, ectopic origin is the most common, while an anomalous course supplying the left ventricular apex is extremely rare. These anomalies are not always benign, and simplistic anatomical approaches to diagnosis and treatment are insufficient. Tailored care for each patient is essential. Future research should focus on understanding the link between these anomalies and myocardial ischemia and on accurately assessing individual risk.
